# Effectiveness of herbal medicine for liver cancer treatment as revealed by a bibliometric and visualization analysis

**DOI:** 10.3389/fonc.2025.1527091

**Published:** 2025-05-08

**Authors:** Yusha Shi, Juwei Wang, Yahui Zhang, Kai Wu, Yibo Zhu, Kaiwen Yan, Qin Ouyang

**Affiliations:** Wenzhou TCM Hospital of Zhejiang Chinese Medical University, Wenzhou, China

**Keywords:** bibliometrics, CiteSpace, VOSviewer, liver cancer, herbal medicine

## Abstract

**Background:**

Liver cancer is highly prevalent worldwide. However, current medical treatments remain insufficient. Although herbal medicine has a long history and extensive expertise in treating liver cancer, the literature in this field has not been thoroughly explored. This study aims to assess and analyze the distribution patterns and key research areas of publications concerning herbal medicine for liver cancer.

**Methods:**

Literature on herbal medicine and liver cancer published between January 1, 2008, and September 28, 2024, was collected for this research. Excel, CiteSpace 6.4.R1, VOSviewer 1.6.20, Scimago Graphica, and Bibliometrix 4.1 were used for data analysis.

**Result:**

The study examines 634 academic articles on herbal medicine for liver cancer, with the majority contributed by Chinese researchers, particularly from Shanghai University of Traditional Chinese Medicine. Wang Ning is the most productive author, possessing the highest h-index. The JOURNAL OF ETHNOPHARMACOLOGY has the most publications and the highest h-index. Journals publishing on herbal medicine and liver cancer are primarily in the fields of molecular biology and immunology, whereas the cited journals are mainly in the fields of environment, toxicology, and nutrition. Keyword clustering analysis indicates that “NF kappa B” and apoptosis have long been the main research topics in this field. Analysis of emergent words suggests that “network pharmacology”, antioxidants, “adjuvant therapy”, and “molecular docking” may become significant research topics in the near future.

**Conclusion:**

This analysis provides a comprehensive overview of the current status, primary focuses, and emerging trends in research related to herbal medicine and liver cancer.

## 1 Introduction

Liver cancer is the fourth most common cause of death globally, resulting in over 800,000 deaths annually (1). The majority of primary liver cancers are hepatocellular carcinoma, accounting for about 90%, followed by intrahepatic cholangiocarcinoma and other primary liver malignancies (2). China alone accounts for 45.3% of the world’s liver cancer cases and 47.1% of liver cancer deaths (3). The hepatitis B virus remains the primary cause of liver cancer mortality, followed by the hepatitis C, alcohol consumption, and noncoholic steatohepatitis (4, 5). Available treatments include surgical resection, transarterial chemoembolization, and ablation (6). However, these do not significantly improve patient survival or alleviate discomfort. Therefore, more effective treatment strategies need to be identified.

Herbal medicine has shown promise in treating liver cancer (7). Modern research indicates that herbs not only affect tumor cells’ growth, proliferation, apoptosis, invasion, and migration, but also alleviate symptoms, reduce side effects after surgery or chemoembolization, and improve patient quality of life and survival (8–10). Herbal therapy could provide additional treatment options and enhance healthcare delivery for liver cancer patients.

Bibliometrics, using mathematical and statistical methods, quantitatively assesses knowledge vectors in different research areas. Its applications include analyzing the evolutionary potential of research areas, the dynamics of knowledge structures, the intensity of collaborations, identifying research hotspots, and predicting development trajectories (11). For example, Liu JM (12) used bibliometrics to study the evolutionary patterns of CDK7 inhibitors in cancer treatment, while Zhao WJ (13) analyzed research trends in COVID-19 acute kidney injury.

Studies have been conducted to investigate the molecular mechanisms and clinical applications of herbal medicine against liver cancer (14, 15). However, the spatial and temporal distribution characteristics, collaborative network relationships, and research hotspots of studies related to herbal medicine and liver cancer on a global scale remain unresolved. This study fills the above gaps through bibliometrics to provide a comprehensive understanding of the distribution characteristics of publications and research hotspots between 2008 and September 28, 2024 on herbal medicines for hepatocellular carcinoma. It also provides personal predictions and insights on several potential hotspots. This study provides data-driven decision-making for interdisciplinary research.

## 2 Materials and methods

### 2.1 Data sources and retrieval strategy

Materials were collected from the Web of Science Core Collection (WoSCC) database and Science Citation Index Expanded (SCI-E) from 2008 to the present. The WoSCC and SCI-E remain indispensable tools for bibliometric research, distinguished by their discipline-wide coverage, high quality data, in-depth citation capabilities and historical integrity. The WoSCC can provide indicators such as H-index, citation count, and journal impact factor. And it is seamlessly integrated with multiple platforms to support visualization and analysis of collaboration networks and research trends. SCI-E focuses on natural science fields, and its selection criteria are more stringent to ensure the academic standardization of the included literature.

We used the following search terms: TS=((“liver cancer” OR “Liver Neoplasm” OR “liver tumors” OR “Hepatocellular Cancers” OR “Hepatoma” OR “Liver Carcinoma” OR “Hepatic tumor” OR “Hepatic cancer” OR “Cancer of Liver” OR “cancer of the liver” OR “hepatic neoplasms” OR “Neoplasm of liver” OR “liver and intrahepatic bile duct carcinoma” OR “liver and intrahepatic biliary tract cancer” OR “Tumor of liver” OR “liver malignant tumors” OR “Neoplasm of the liver” OR “Hepatocellular Carcinoma”) AND (“herbal medicine” OR “herbal therapy” OR “herb therapy” OR “phytotherapy” OR “drugs, Chinese herbal” OR “ethnobotany”)). The search was completed on September 28, 2024, including 799 publications.

### 2.2 Data extraction and processing methods

To ensure our inclusion criteria were met, we carefully reviewed the type and full texts of the search results. Publications were screened according to the following inclusion and exclusion criteria:

**Inclusion Criteria:**

1. The research topics of the publications are liver cancer and herbal medicine.
2. Document types are Articles and review articles.
3. Literature published in English.
4. A portion of the publications addresses liver cancer and herbal medicine, with substantial content covering at least one.
5. Literature not directly discussing liver cancer, herbs, needs to be clearly focused on at least one of the following areas: possible pathogenic mechanisms, precursor diseases, signaling pathways, etc., of herbal medicines in treating liver cancer.

**Exclusion Criteria:**

1. The research topics and content of the publications are unrelated to liver cancer and herbal medicine.
2. Literature published in Non-English.
3. Although some portions of the publications cover liver cancer and herbal medicine, the content is minimal, typically comprising only a few quotations or statements without in-depth discussion.
4. Articles are incomplete or only summaries are available.
5. Duplicate publications.

**Figure 1:**
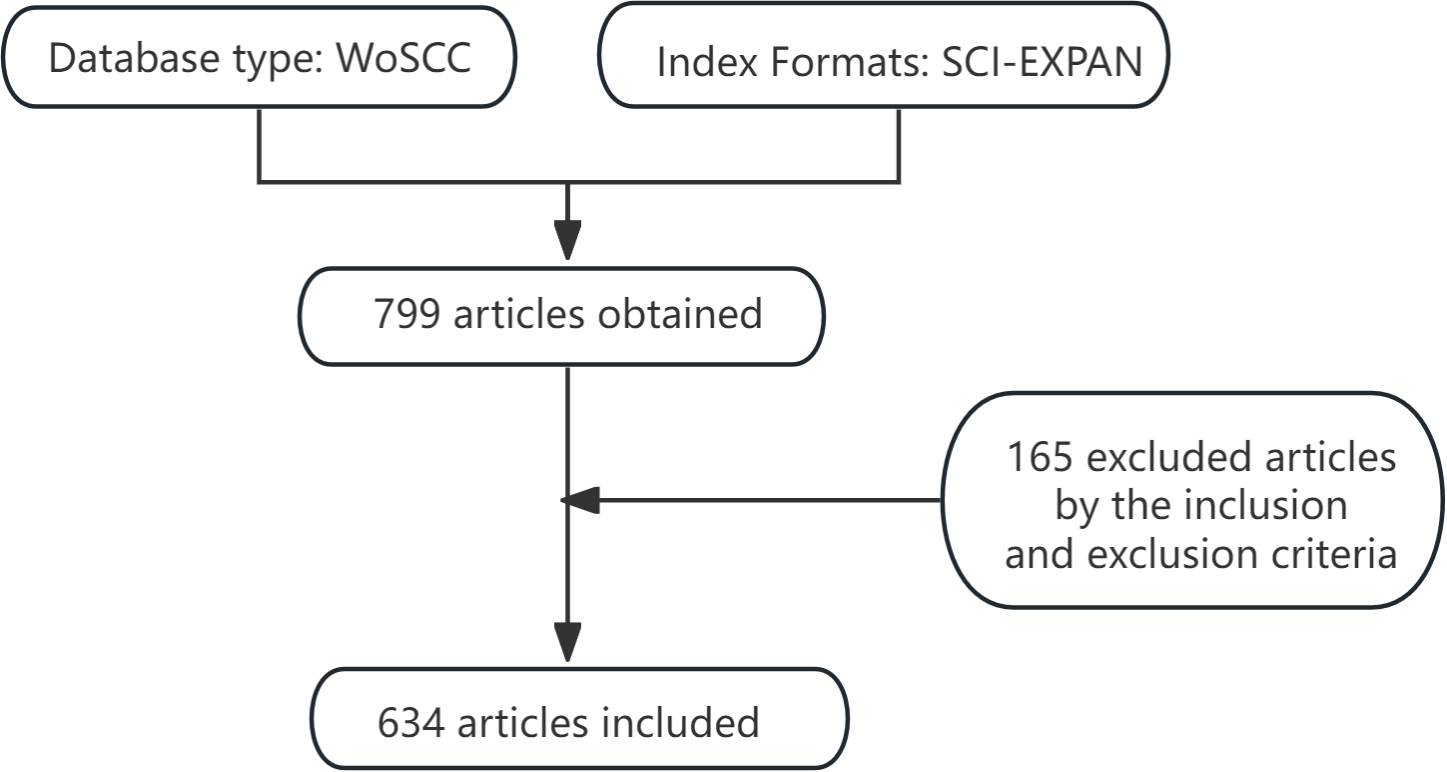
Article search and screening process.

After the scrutiny, we obtained a total of 634 documents (**Figure 1**).

### 2.3 Analysis software

The data did not contain identifiable patient information and therefore did not require an ethical review. Excel, Citespace 6.4.R1, VOSviewer 1.6.20, Scimago Graphica, and Bibliometrix 4.1 were used for data analysis (16).

The annual volume of publications is summarized in Excel. Analyze countries, institutions, authors, journals, article co-citations, and keywords using Citespace 6.4.R1. Utilize Scimago Graphica and VOSviewer 1.6.20 to create country cooperation network maps. Analyze authors and journals using Bibliometrix 4.1.

### 2.4 Interpretation of main parameters

The Hirsch index (H-index) indicates that at least H published papers have been cited at least H times (17). It is a useful measure for evaluating the volume and impact of a researcher’s scholarly output. A higher H-index indicates greater contribution and influence. Centrality serves as a measure of the impact of nodes within a network. Nodes with higher centrality are connected to more other nodes. Burst analysis can reveal abrupt shifts in citations or keywords during a specified time frame, helping to identify important nodes and gain insights into emerging research trends. In the graph, node size indicates the number of publications, colors indicate different clusters, and line thickness indicates the degree of cooperation.

## 3 Results

### 3.1 Annual publication number analysis

**Figure 2:**
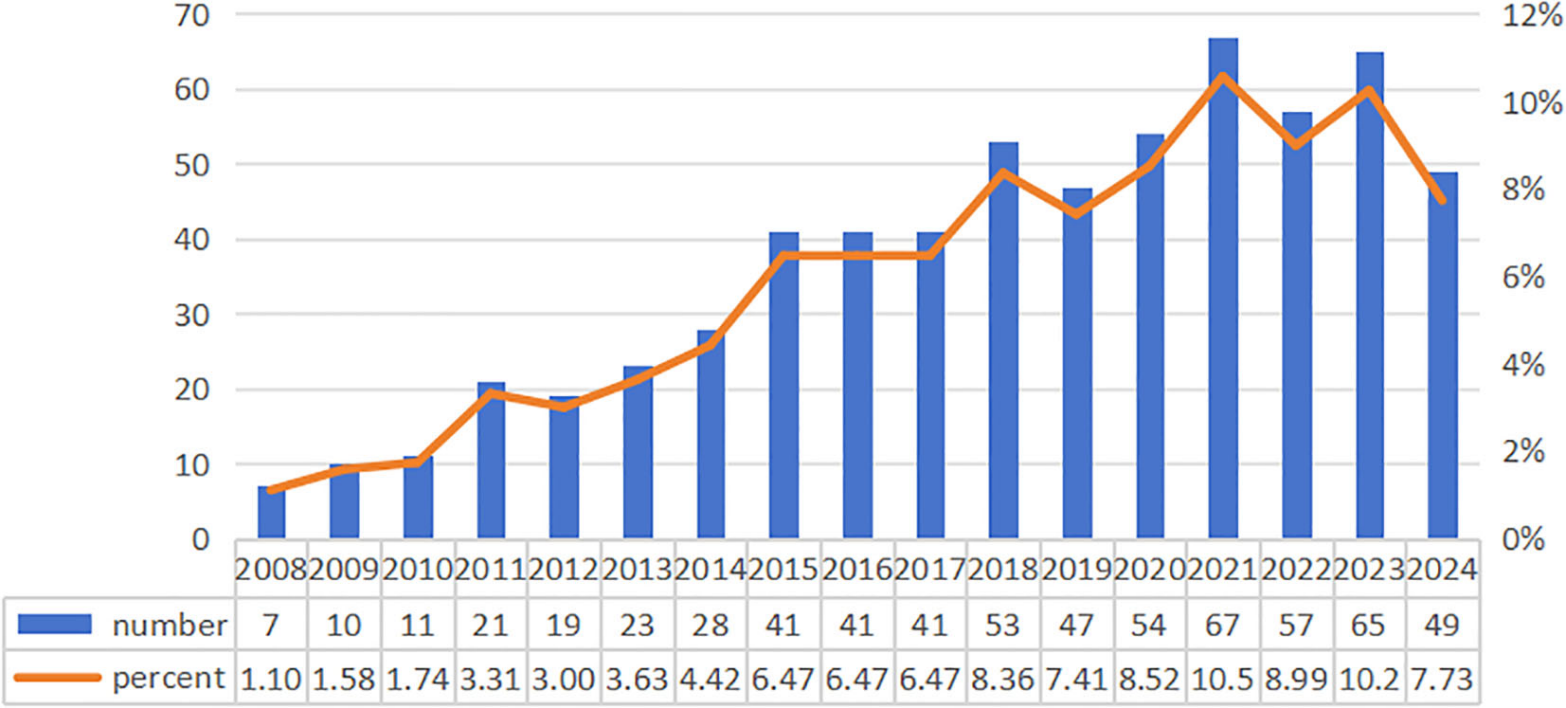
Annual number of published papers. The horizontal axis represents the time of publication. The blue bar graph represents the annual publication number, corresponding to the value on the left. The orange line graph represents the percentage of the annual publication number, corresponding to the values on the right.

The study encompasses an analysis of 634 scholarly articles about herbal medicine for liver cancer. As shown in **Figure 2**, the highest publication year was 2021 (67) and the lowest was 2008 (7). The annual growth rate for publications is 12.93%.

### 3.2 Country analysis

**Figure 3:**
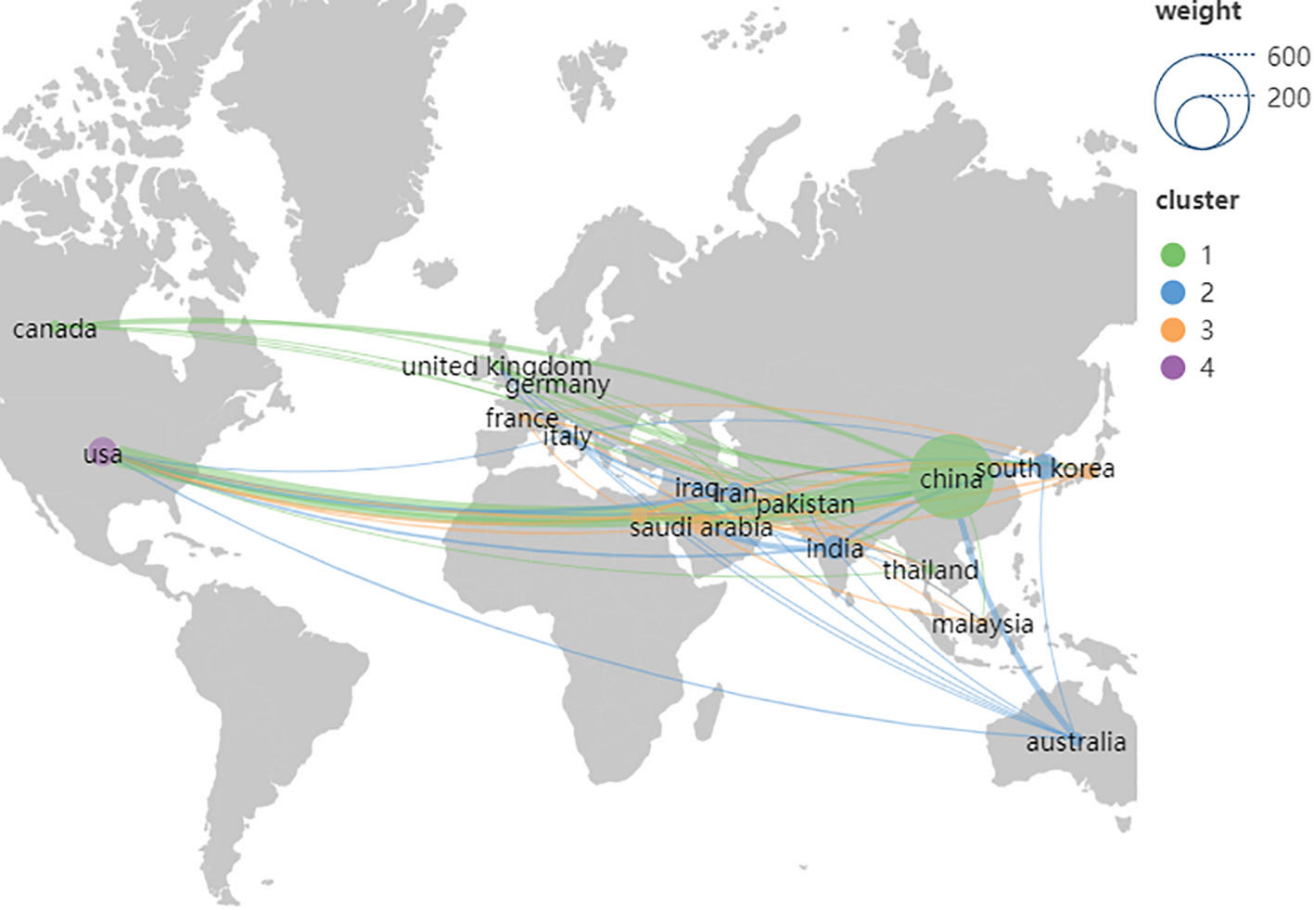
The map of the top 18 countries’ cooperation networks. The node size indicates the number of publications, colors indicate different clusters, and lines indicate partnerships. The thicker the line between the connecting points, the closer the cooperation between the two countries.

A total of 61 countries contributed to research on herbal medicine and liver cancer. **Table 1** lists the top ten countries by the number of publications. China led with 456 articles, accounting for 53.52% the total. The United States followed with 53 publications, South Korea with 43, India with 37, and Saudi Arabia with 25. China, the USA, and India stood out with higher centrality values (0.52, 0.35, and 0.25, respectively), indicating their pivotal roles in international cooperation. Despite a low publication count (8), the United Kingdom ranked fourth in centrality (0.16), showcasing its high-quality publications and extensive scientific cooperation with other countries. **Figure 3** illustrates the collaborative networks of the top 18 countries by publication count. **Figure 3** indicates that China maintains a high level of cooperation and exchange with the United States and Australia.

**Table 1:** The top 10 countries in terms of publications.

Rank	Countries	Publications	Centrality
1	China	456	0.52
2	USA	53	0.35
3	South Korea	43	0.15
4	India	37	0.25
5	Saudi Arabia	25	0.06
6	Iran	22	0.09
7	Japan	17	0.01
8	Egypt	17	0.05
9	Pakistan	12	0.07
10	Australia	12	0.1

### 3.3 Institutions analysis

**Figure 4:**
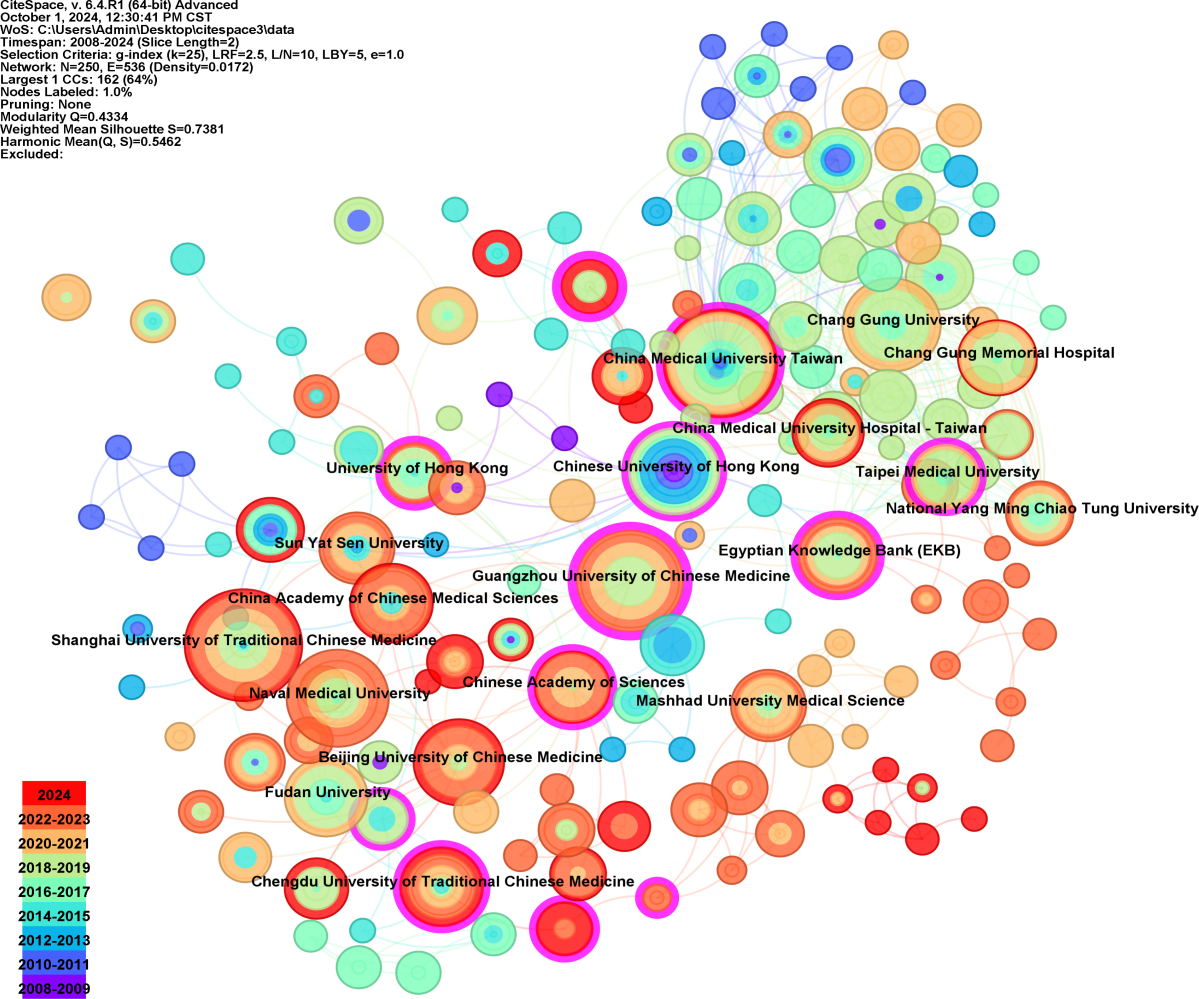
The collaboration network of institutions. The node size indicates the number of publications of the institution. The color of the node corresponds to the annual publication time. The light purple ring on the outermost ring indicates high centrality of the node. (Figure created using CiteSpace).

**Table 2** highlights the significant contributions of Chinese research institutions in the field. Shanghai University of Traditional Chinese Medicine leads with 29 publications, followed by China Medical University Taiwan with 28 and Guangzhou University of Chinese Medicine with 22. Despite its relatively low global publication count, Guangzhou Medical University surpasses other institutions in centrality (n=0.24), indicating widespread recognition of its research. The Egyptian Knowledge Bank has published 16 articles, the highest number outside Chinese research institutions. **Figure 4** presents a co-occurrence graph of publishing.

**Table 2:** The top 10 institutions in terms of publications.

Rank	Institutions	Publications	Centrality	Country
1	Shanghai University of Traditional Chinese Medicine	29	0.07	China
2	China Medical University Taiwan	28	0.14	China
3	Guangzhou University of Chinese Medicine	22	0.23	China
4	Naval Medical University	19	0.01	China
5	Beijing University of Chinese Medicine	18	0.04	China
6	Chang Gung University	17	0.08	China
7	Egyptian Knowledge Bank	16	0.13	Egypt
8	Fudan University	15	0.04	China
9	China Medical University Hospital - Taiwan	15	0.06	China
10	Chinese Academy of Sciences	14	0.1	China

### 3.4 Authors analysis

**Figure 5:**
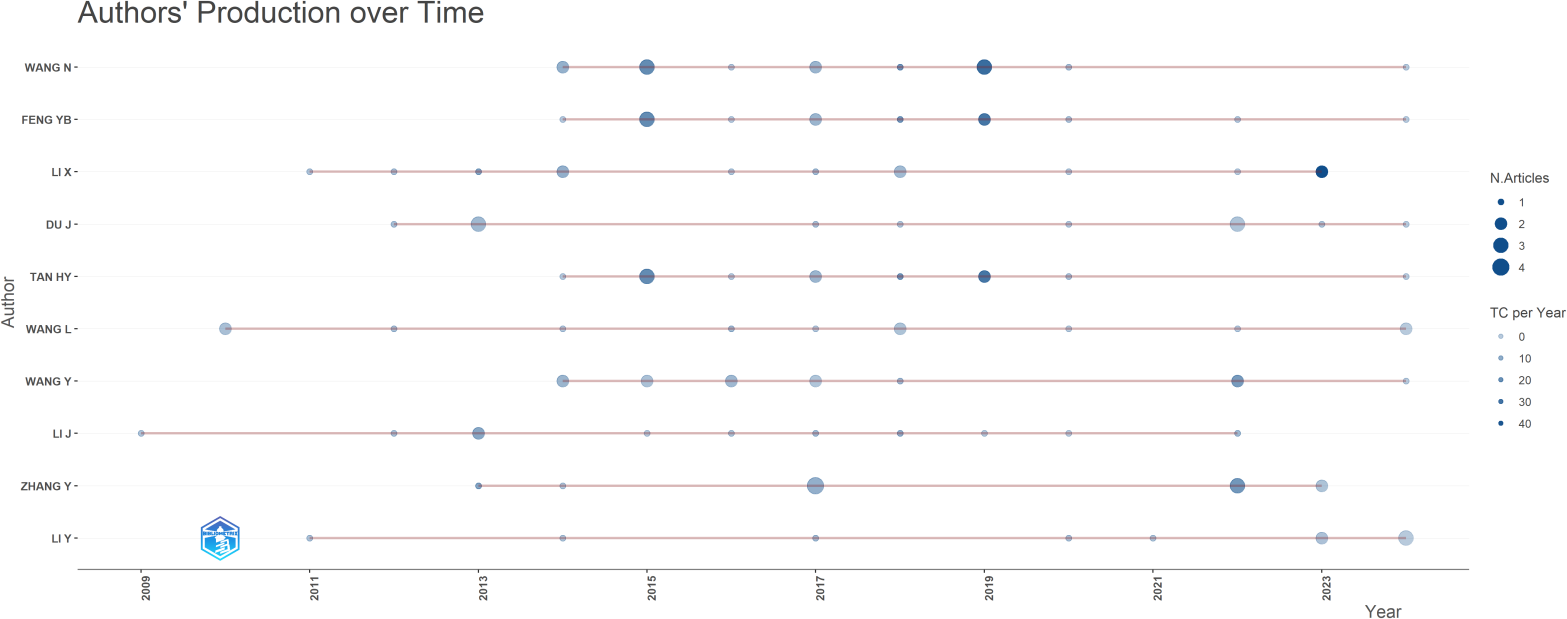
The achievements of authors over time. The size of the nodes indicates the number of articles published by the author. The color indicates the number of citations, and the darker the color, the more citations.

**Figure 6:**
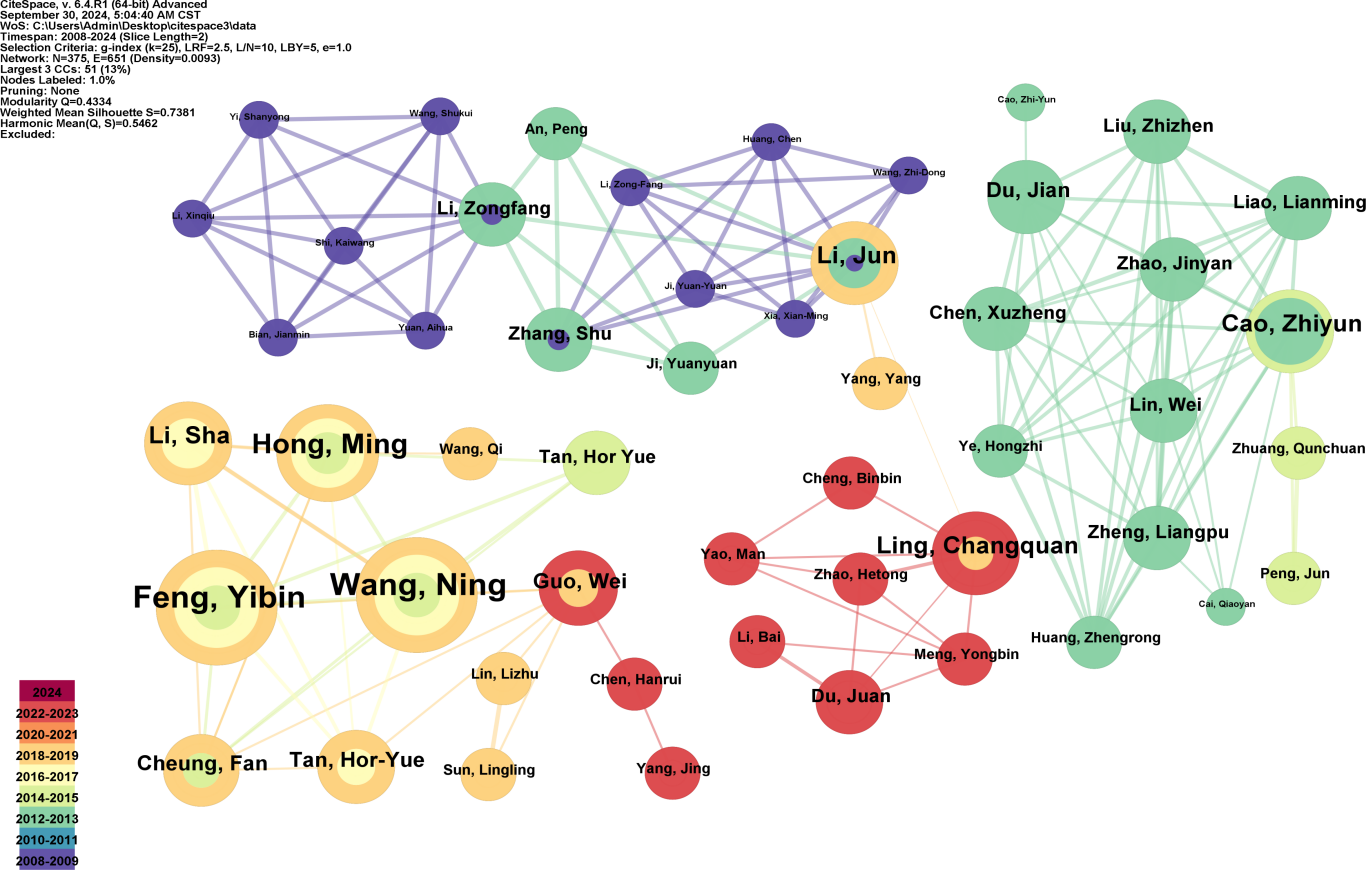
The collaboration network of authors. The size of the nodes indicates the number of articles published by the author. The lines between the nodes represent the collaborative relationships. The color corresponds to the annual publication time period from 2008 to 2024. (Figure created using CiteSpace).

A total of 375 authors contributed to research on herbal medicine and liver cancer during the study period. **Table 3** lists the top ten most productive authors, all from China. It is regrettable that no non-Chinese scholars have appeared on the list thus far. Wang Ning ranks as the most prolific author with 14 articles, followed by Feng YiBin and Li Xin, who each have 13 articles. They also possess high h-indices, indicating their influence in the field. **Figure 5** illustrates the authors’ achievements over time, while **Figure 6** displays an author collaboration network constructed using CiteSpace.

**Table 3:** The top 10 most productive authors.

Rank	Author	Publications	h_index	Citations
1	Wang Ning	14	11	718
2	Feng Yibin	13	10	618
3	Li Xin	13	10	535
4	Tan Horyue	12	10	613
5	Wang Ying	12	9	270
6	Du Jian	12	7	181
7	Wang Lu	12	6	188
8	Li Jun	11	8	395
9	Zhang Yu	11	8	308

### 3.5 Journals analysis

**Figure 7:**
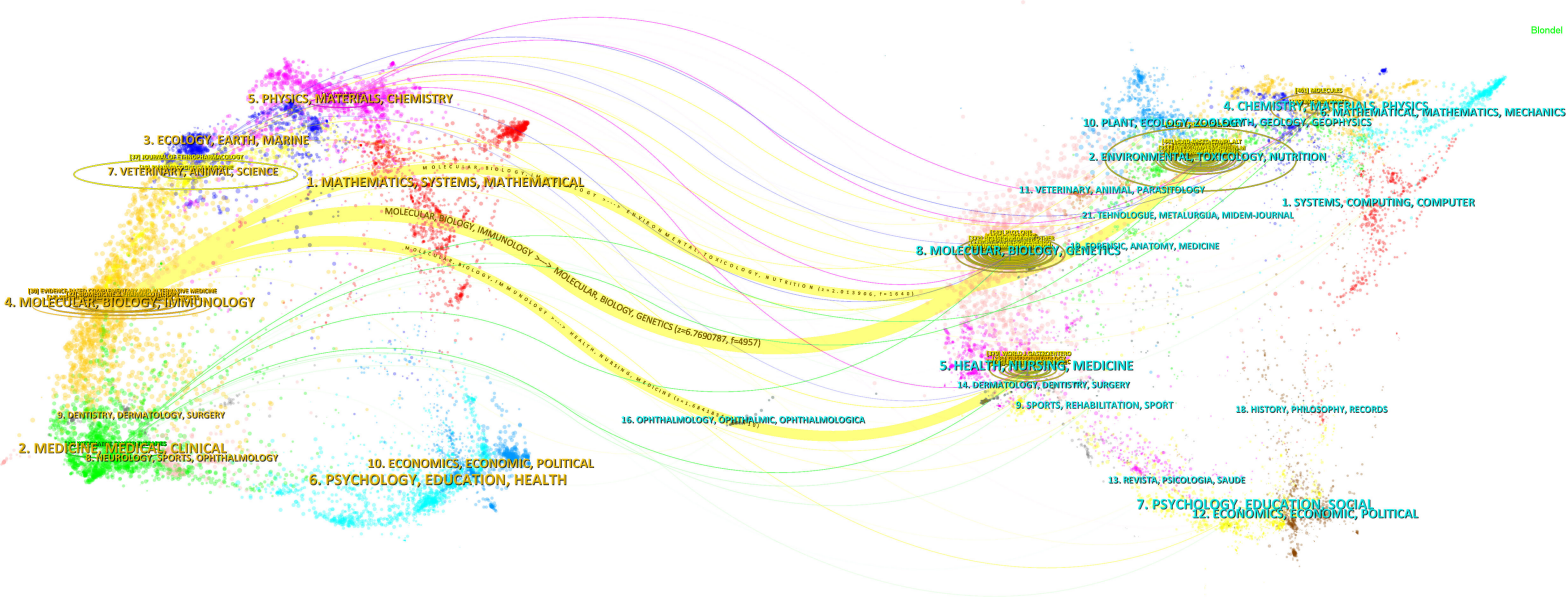
The dual-map overlay. The dots represent journals, with journals citing references on the left and cited journals on the right. The curve between the left and right side indicates the citation link. The weight assigned to a color for a particular cluster is determined by the number of items in the neighborhood of that point that belong to that cluster, with each color representing each cluster.

Throughout the study period, 259 journals published articles on herbal medicine and liver cancer. **Table 4** displays the top ten journals, with the JOURNAL OF ETHNOPHARMACOLOGY leading in publications, followed by EVIDENCE-BASED COMPLEMENTARY AND ALTERNATIVE MEDICINE and BIOMEDICINE & PHARMACOTHERAPY. BIOMEDICINE & PHARMACOTHERAPY boasts the highest impact factor (n=6.9). Despite having a relatively small number of publications, ONCOLOGY REPORTS possesses a high h-index, indicating high-quality literature. The dual-map overlay in **Figure 7** illustrates that journals publishing on herbal medicine and liver cancer are primarily in the fields of molecular biology and immunology, whereas the cited journals are mainly in the fields of environment, toxicology, and nutrition.

**Table 4:** The top 10 most productive journals.

Rank	Source	h_index	Citations	Publications	IF
1	JOURNAL OF ETHNOPHARMACOLOGY	16	922	37	4.8
2	EVIDENCE-BASED COMPLEMENTARY AND ALTERNATIVE MEDICINE	13	471	30	2.65
3	ONCOLOGY REPORTS	13	360	14	3.8
4	BIOMEDICINE & PHARMACOTHERAPY	12	454	22	6.9
5	INTERNATIONAL JOURNAL OF MOLECULAR SCIENCES	10	518	14	4.9
6	MOLECULES	10	451	12	4.2
7	INTEGRATIVE CANCER THERAPIES	9	224	15	2.9
8	PHYTOTHERAPY RESEARCH	9	260	12	6.1
9	FRONTIERS IN PHARMACOLOGY	8	304	19	4.4
10	PHYTOMEDICINE	8	447	15	6.7

### 3.6 Article co-citation analysis

Small (18) first introduced the concept of co-citation as a research method to assess the degree of relationship between documents in 1973. The number of citations can reflect the impact of an article in a particular field of research. **Table 5** lists the ten papers with the highest co-citation strengths. The most prevalent article type is the multicenter study. According to CiteSpace analysis, the publication with the highest co-citation strength and total citations is “Global Cancer Statistics 2020: GLOBOCAN Estimates of Incidence and Mortality Worldwide for 36 Cancers in 185 Countries” (19). The article, authored by Sung H and published in the A Cancer Journal for Clinicians in 2021, appears in the journal with the highest impact factor in the world. It presents an updated analysis of the global cancer burden using the GLOBOCAN 2020 estimates of cancer incidence and mortality provided by the International Agency for Research on Cancer.

**Table 5:** The top 10 papers with the highest cocitation strength.

Rank	Cocitation strength	Total citation	Title	Categories
1	9.38	36	Global Cancer Statistics 2020: GLOBOCAN Estimates of Incidence and Mortality Worldwide for 36 Cancers in 185 Countries	Multicenter Study
2	5.4	13	Cancer incidence and mortality worldwide: sources, methods and major patterns in GLOBOCAN 2012	Multicenter Study
3	5.22	9	Thyroid cancer management: from a suspicious nodule to targeted therapy	Review
4	4.46	13	A global view of hepatocellular carcinoma: trends, risk, prevention and management	Review
5	4.33	10	Traditional herbal medicine prevents postoperative recurrence of small hepatocellular carcinoma: A randomized controlled study	Randomized Controlled Trial
6	3.92	10	Chinese herbal medicine-derived compounds for cancer therapy: a focus on hepatocellular carcinoma	Review
7	3.89	11	Global cancer statistics, 2012	Multicenter Study
8	3.83	11	Lenvatinib versus sorafenib in first-line treatment of patients with unresectable hepatocellular carcinoma: a randomized phase 3 non-inferiority trial	Clinical Trial
9	3.8	13	Cancer statistics in China, 2015	Multicenter Study
10	3.61	7	Hepatocellular carcinoma	Commet

### 3.7 Keywords analysis

**Figure 8:**
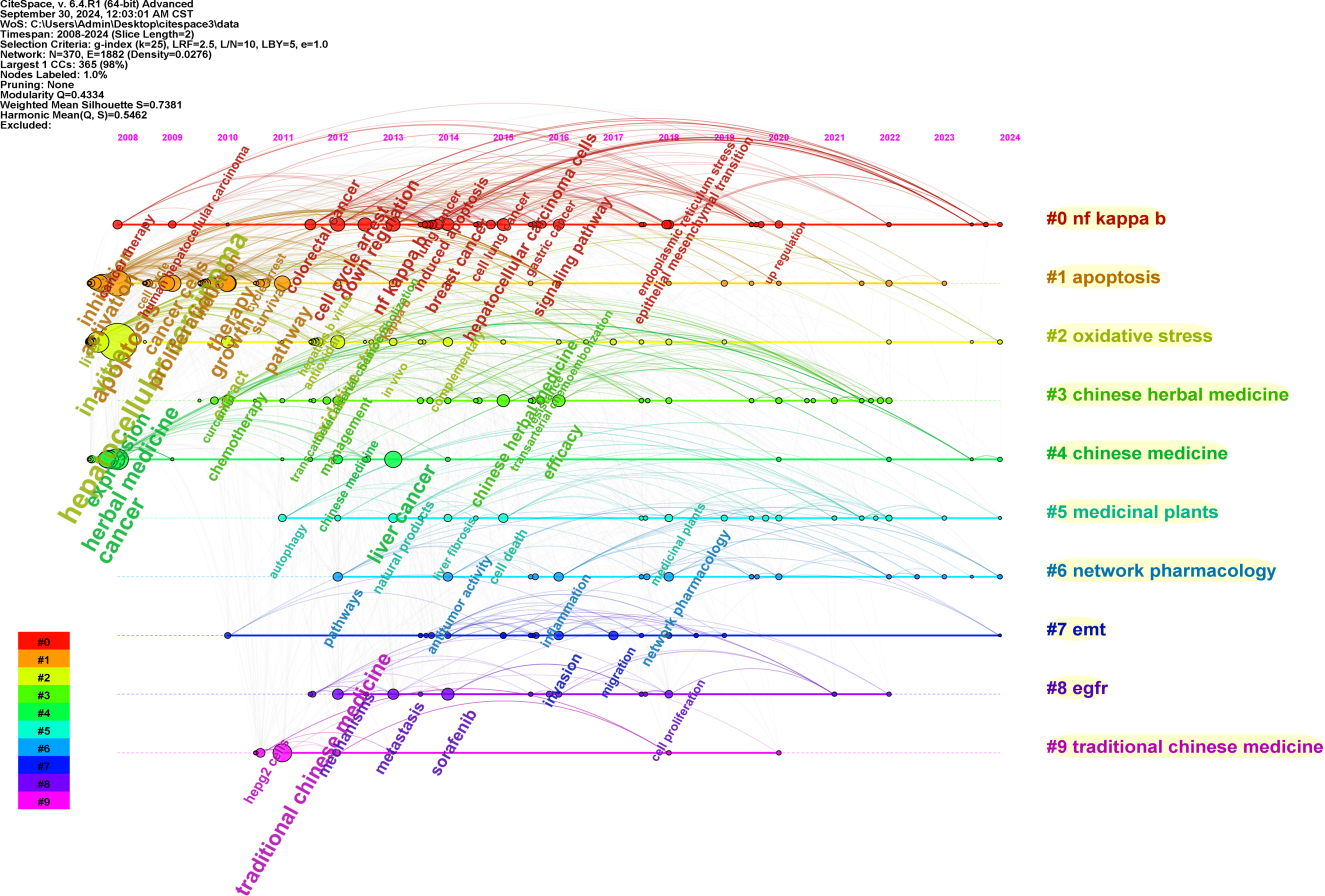
Timeline view of keyword clustering analysis. The size of the node represents how often the cited literature is cited. The horizontal axis represents when the literature was published. The lines between the nodes represent the collaborative relationships. The cluster labels (right) summarize major research themes in the field. (Figure created using CiteSpace).

**Figure 9:**
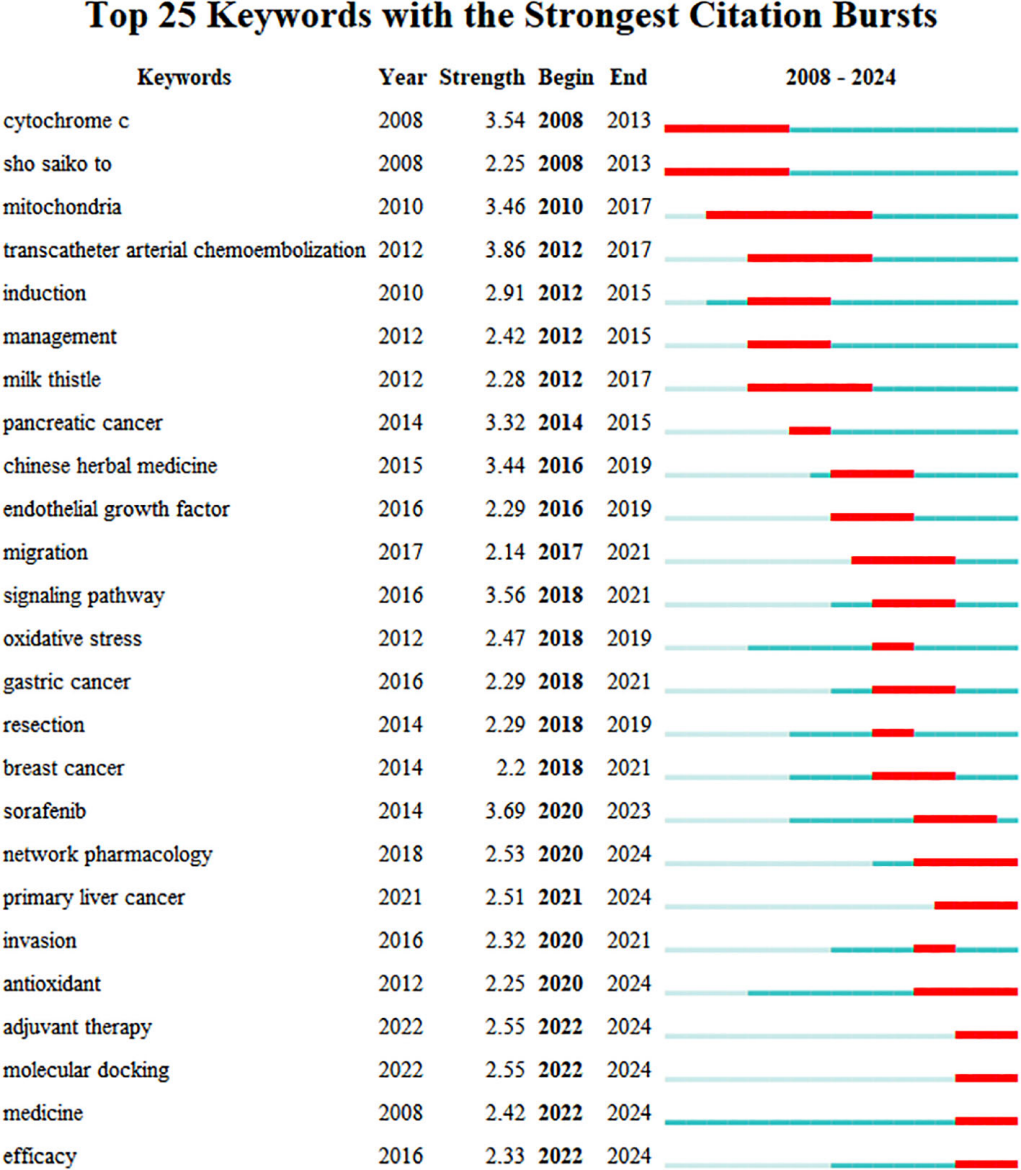
The top 25 keywords with the strongest citation bursts. The blue line indicates the timeline, and the red bar indicates the burst period.

Keywords are essential for reflecting the main topics of an article. **Table 6** showcases the ten most frequently used keywords in the study, with “hepatocellular” (n=307), “carcinoma” (n=142), and “*in vitro*” (n=93) being the most prevalent. The timeline view of keyword clustering created by CiteSpace (**Figure 8**) highlights specific clusters such as “NF kappa B”, apoptosis, “oxidative stress”, “Chinese herbal medicine”, “Chinese medicine”, and “medicinal plants” with respective sizes of 82, 68, 67, 54, 45, and 42. These categories represent the primary classifications in herbal medicine related to liver cancer. **Figure 9** illustrates the top 25 keywords with the strongest citation bursts. “Network pharmacology”, antioxidant, “adjuvant therapy”, and “molecular docking” are poised to emerge as significant areas of research in the near future.

**Table 6:** The top 8 keywords with the highest frequency.

Rank	Keywords	Frequency
1	hepatocellular carcinoma	307
2	apoptosis	142
3	*in vitro*	93
4	cancer	91
5	herbal medicine	83
6	traditional Chinese medicine	67
7	expression	64
8	activation	63

## 4 Discussion

### 4.1 General information

In recent years, a growing body of literature has demonstrated the efficacy of herbal medicine in treating liver cancer. China has emerged as a leader in this field, with the highest volume and centrality of publications, and the top 10 most productive authors all coming from Chinese research institutions. The top 10 publications on herbal medicine for liver cancer were all affiliated with Chinese research institutions. All of the aforementioned analyses suggest that herbal medicine for liver cancer exhibits a more distinct geographical character. This creates specific challenges for the global dissemination of the discipline’s culture. These publications appeared in 259 different journals, with the Journal of Ethnopharmacology having the highest number of publications and the highest h-index. The journals publishing articles on herbal medicine for liver cancer primarily focused on molecular biology and immunology, while the cited journals were predominantly from the fields of environment, toxicology, and nutrition.

### 4.2 Hotspots and frontiers

We used CiteSpace to conduct a keyword analysis to assess current research trends and frontiers. The findings revealed that the most frequently occurring keywords from 2008 to 2024 were hepatocellular carcinoma, apoptosis, and “*in vitro*”. Additionally, our analysis identified “NF kappa B” and apoptosis as the leading keyword clusters in current research. Based on the assessment of trending keywords, “network pharmacology”, antioxidants, “adjuvant therapy”, and “molecular docking” are anticipated to emerge as popular research areas in the coming years.

#### 4.2.1 Adjunctive therapy

Herbal medicine complements conventional treatments by Surgery, ablation, and transarterial chemoembolization are the primary treatment modalities for liver cancer (6). These interventions aim to eliminate or destroy cancer cells. However, these treatments are associated with potential complications, toxicity, and drug resistance. Herbal medicine is used as a supplementary approach in managing liver cancer (20). It has been shown to enhance the effectiveness of primary treatments and reduce adverse side effects (21). A study at Beijing Ditan Hospital of Capital Medical University suggests that using traditional Chinese medicine as an adjunctive therapy may extend median survival time and improve overall survival in patients with HCC (22). Research by Luo Ru (23) indicates that using Jianpi Huayu decoction as a complementary treatment for HCC following hepatectomy is associated with a low rate of postoperative recurrence and high overall survival. Incorporating herbal remedies as adjunctive therapy in managing liver cancer has significantly improved patient prognosis. Some studies have shown the potential adjunctive role of herbal therapy in addressing tumor cell metabolism (24). In addition to traditional compound herbs, single herbs have become a hot research topic in the adjuvant treatment of hepatocellular carcinoma. Single herbs and plant derivatives such as Crithmum maritimum and Crithmum maritimum L can reduce amino acid levels and prevent and improve HCC by lowering choline, phosphorylcholine, and regulating lipid homeostasis (25, 26). These emerging complementary therapies are promising and worthy of attention in clinical applications.

#### 4.2.2 Antioxidant

A strong correlation exists between oxidative stress and the development of liver cancer (27–29). Hepatocytes are crucial in producing oxygen-free radicals in the body. When liver cell function is compromised or metabolic imbalance occurs, a large quantity of oxidative free radicals is produced, leading to intracellular oxidative stress. The inability to clear these free radicals can ultimately lead to the development of liver cancer. Cancer progression is driven by the imbalance between pro-oxidants, such as reactive oxygen species (ROS), and endogenous antioxidant molecules. Herbal medicine, a key component of traditional medicine in many cultures, serves as a natural antioxidant (30). It is rich in flavonoids, phenolic compounds, saponins, and active polysaccharides (31). Natural antioxidants play a crucial role in treating liver diseases caused by free radicals by alleviating oxidative stress and counteracting the effects of these radicals (32). The use of natural antioxidants is becoming increasingly significant in managing liver cancer. In recent years, antioxidant therapy for hepatocellular carcinoma has attracted significant attention from scholars. Studies have demonstrated that extracts from Coleus and Liuwei Dihuang Pills may have anticancer potential in hepatocellular carcinoma by modulating ROS levels and mitochondrial membrane potential (33, 34). Numerous studies have demonstrated the antioxidant effects of herbs in treating liver cancer.

#### 4.2.3 Molecular docking

Molecular docking studies the binding affinity between protein receptors and small drug molecules at the molecular level, describing their conformational relationships (35). Unlike traditional research methods, molecular docking employs computer software to identify potential key active substances that interact with target proteins through geometric and energy matching based on the lock-and-key principle (36). Molecular docking predicts drug-target interactions and is widely applied in natural product research. For example, Shehawy demonstrated that the strong interaction between the herbal active ingredients Thymoquinone and Piperine with DNMT3B and HDAC3 inhibited the activity of hepatocellular carcinoma cells and led to growth arrest and cell death by molecular docking (37). It provides a theoretical basis for elucidating mechanisms of action and offering novel insights into the study of traditional herbal medicine.

Emerging applications of molecular docking technology include predicting adverse reactions, drug repurposing, pharmacology, and target fishing and profiling (38). In recent years, molecular docking has gained prominence in exploring active ingredients in herbal remedies. For example, Tang (39) verified the mechanism of anti-HCC of Polygonum perfoliatum L extraction, a traditional Chinese medicine plant. Molecular docking simulations were conducted to investigate the binding affinities of ADRA1B, PLCB2, PRKG2, and GLO1 as protein receptors for the compounds of interest.

#### 4.2.4 Network pharmacology

Network pharmacology is an interdisciplinary field that emerges from the integration of biology and computer technology. By utilizing high-throughput genomics data analysis, computer simulations, and open database searches, network pharmacology seeks to uncover the complex network relationships involved in drug-gene-target-disease interactions (40). The concept of network pharmacology was first introduced by the British pharmacologist A. L. Hopkins in 2007 (41). Network pharmacology is characterized by its “multi-component, multi-target, and multi-channel” features. Compared to traditional pharmacological experiments, network pharmacology provides unique advantages in preventing and treating complex diseases. Network pharmacology increases the success rate of new drug clinical trials, and reduces the costs of drug research and development (42). In recent years, network pharmacology has attracted significant attention from scholars. Interestingly, numerous articles used network pharmacology combined with molecular docking techniques, incorporating experimental validation to further confirm the mechanisms of action and efficacy of drugs, thereby enhancing the reliability and accuracy of prediction results (43). The investigation of herbal treatments for hepatocellular carcinoma has become a focal area of research. The diverse active components found in herbs and the complex interactions between these components merit investigation. For instance, Jihan Huang (44) predicted that ten compounds, including betaine, epoetin, and berberine, derived from the herbal remedy Xianglian pill, exhibit activity against multiple HCC-related targets and pathways in the treatment of hepatocellular carcinoma through the integration of network pharmacology and RNA sequencing.

#### 4.2.5 Strengths and limitations

This study offers several significant advantages. Firstly, it provides a comprehensive and systematic bibliometric analysis of herbal remedies for hepatocellular carcinoma, addressing a notable gap in the existing literature. Secondly, our data analysis methodology is highly objective, employing widely recognized bibliometric tools such as CiteSpace, VOSviewer, and Bibliometrix. Finally, we provide multiple new perspectives on molecular research to clinical citations. This helps to understand the current state of research and identify future research hotspots.

However, there are certain limitations to this study. Firstly, the data utilized in this study were exclusively sourced from the WoSCC database and SCIE (2008-present), thereby disregarding valuable information from other databases and indexing methods. Secondly, Publications predating 2008 were excluded from our analysis due to limited availability of historical data. Data were limited to publications before September 2024, which may affect the accuracy of the prediction of emerging hotspots. Thirdly, only articles, reviews, and English-language literature were included. This potentially resulted in an incomplete literature collection, overlooked the importance of non-English articles, and led to an incomplete presentation of geographical research trends. Lastly, the absolute dominance of Chinese institutions may mask small-scale innovative research in other countries, which needs to be interpreted in the context of regional policy and financial support.

## 5 Conclusions

This study analyzes the evolution of the field of herbal medicine for liver cancer from 2008 to September 28, 2024. China has made the most significant contributions to this field and ranks first in the number of published papers, authors, and institutions. Shanghai University of Traditional Chinese Medicine is the leading institution in terms of publication output. Wang Ning is the most prolific author in this field. The JOURNAL OF ETHNOPHARMACOLOGY has published the highest number of articles. “NF kappa B” and apoptosis have historically been the primary research topics in this field. “Network pharmacology”, antioxidants, “adjuvant therapy”, and “molecular docking” are likely to emerge as future research hotspots.

The findings of this study have the following implications for clinical practice and future research. Herbal medicine as adjuvant therapy reveals its gradual emergence in areas such as alternative treatment and prevention of liver cancer, which could be promoted for inclusion in the guidelines for the comprehensive treatment of liver cancer. The antioxidant effect of herbs is very significant, and research focusing on uncovering the antioxidant mechanism of herbs in liver cancer treatment deserves attention. We believe that network pharmacology, molecular docking and other means will continue to be strong in the future, and the combined application between them is already a popular trend. In the future, based on this combination, together with basic experiments, it may become the key project of each subject group.

## Data Availability

The original contributions presented in the study are included in the article/supplementary material. Further inquiries can be directed to the corresponding author.
